# Prion strains viewed through the lens of cryo-EM

**DOI:** 10.1007/s00441-022-03676-z

**Published:** 2022-08-27

**Authors:** Szymon W. Manka, Adam Wenborn, John Collinge, Jonathan D. F. Wadsworth

**Affiliations:** MRC Prion Unit at UCL, Institute of Prion Diseases, University College London, 33 Cleveland Street, London, W1W 7FF UK

**Keywords:** Cryo-EM, Prion, Prion disease, Prion strains, Prion structure

## Abstract

Mammalian prions are lethal transmissible pathogens that cause fatal neurodegenerative diseases in humans and animals. They consist of fibrils of misfolded, host-encoded prion protein (PrP) which propagate through templated protein polymerisation. Prion strains produce distinct clinicopathological phenotypes in the same host and appear to be encoded by distinct misfolded PrP conformations and assembly states. Despite fundamental advances in our understanding of prion biology, key knowledge gaps remain. These include precise delineation of prion replication mechanisms, detailed explanation of the molecular basis of prion strains and inter-species transmission barriers, and the structural definition of neurotoxic PrP species. Central to addressing these questions is the determination of prion structure. While high-resolution definition of ex vivo prion fibrils once seemed unlikely, recent advances in cryo-electron microscopy (cryo-EM) and computational methods for 3D reconstruction of amyloids have now made this possible. Recently, near-atomic resolution structures of highly infectious, ex vivo prion fibrils from hamster 263K and mouse RML prion strains were reported. The fibrils have a comparable parallel in-register intermolecular β-sheet (PIRIBS) architecture that now provides a structural foundation for understanding prion strain diversity in mammals. Here, we review these new findings and discuss directions for future research.

## Introduction

Prions are exceptional lethal pathogens that cause fatal neurodegenerative diseases in mammals. These diseases include Creutzfeldt–Jakob disease (CJD), variant CJD (vCJD), kuru, fatal familial insomnia (FFI), and Gerstmann–Sträussler–Scheinker disease (GSS) in humans, and in animals, bovine spongiform encephalopathy (BSE) in cattle, scrapie in sheep and goats, and chronic wasting disease (CWD) in cervids (Prusiner 1998; Collinge 2001, 2005; Wadsworth and Collinge 2011; Greenlee and Greenlee 2015; Kim et al. 2017; Benestad and Telling 2018). Prions consist of infectious multichain fibrillar assemblies of misfolded host-encoded prion protein (PrP), a glycosylphosphatidylinositol (GPI)-anchored cell surface glycoprotein containing two asparagine (N)-linked glycosylation sites (Prusiner 1998; Wuthrich and Riek 2001; Collinge and Clarke 2007; Collinge 2016; Rodriguez et al. 2017). Prion propagation occurs by means of seeded protein polymerisation, involving recruitment of PrP monomers to existing fibrillar assemblies which elongate and then fragment to generate more “seeds” (Prusiner 1998; Collinge and Clarke 2007; Collinge 2016; Rodriguez et al. 2017; Meisl et al. 2021). Different prion strains produce different disease phenotypes in the same host and appear to be encoded by fibrillar assemblies with distinct misfolded PrP conformations and PrP glycoform assembly states (Collinge et al. 1996; Prusiner 1998; Collinge and Clarke 2007; Wadsworth et al. 2010; Wadsworth and Collinge 2011; Collinge 2016).

While historically rare in humans, the appearance of vCJD in the UK from 1995 onwards (Will et al. 1996; Collinge et al. 1996; Collinge 1999), and the experimental confirmation that this was caused by dietary exposure to BSE prions from cattle (Collinge et al. 1996; Hill et al. 1997; Bruce et al. 1997; Asante et al. 2002), spurred intense international efforts to understand the fundamental basis of prion transmission barriers in order to protect public health (Collinge 1999, 2001; Wadsworth and Collinge 2011). While public and animal health controls to limit human exposure to BSE prions have been highly effective, the existence or emergence of other animal prion strains with zoonotic potential is of considerable concern. In particular, CWD is a contagious disease in free-ranging and captive cervid populations and considerable human exposure may be occurring in North America through consumption of hunted deer (Benestad and Telling 2018; Escobar et al. 2020; Tranulis et al. 2021). In 2016, CWD was also reported in Europe (Benestad and Telling 2018; Tranulis et al. 2021). As it is well known that novel, potentially zoonotic, prion strains with altered host ranges can arise as a result of PrP polymorphisms in both inter- and intra-species transmissions (Collinge and Clarke 2007; Wadsworth et al. 2010; Wadsworth and Collinge 2011; Collinge 2016; Moreno and Telling 2017; Mead et al. 2019), understanding how prion strains are encoded is of high importance (Watson et al. 2021).

**Fig. 1:**
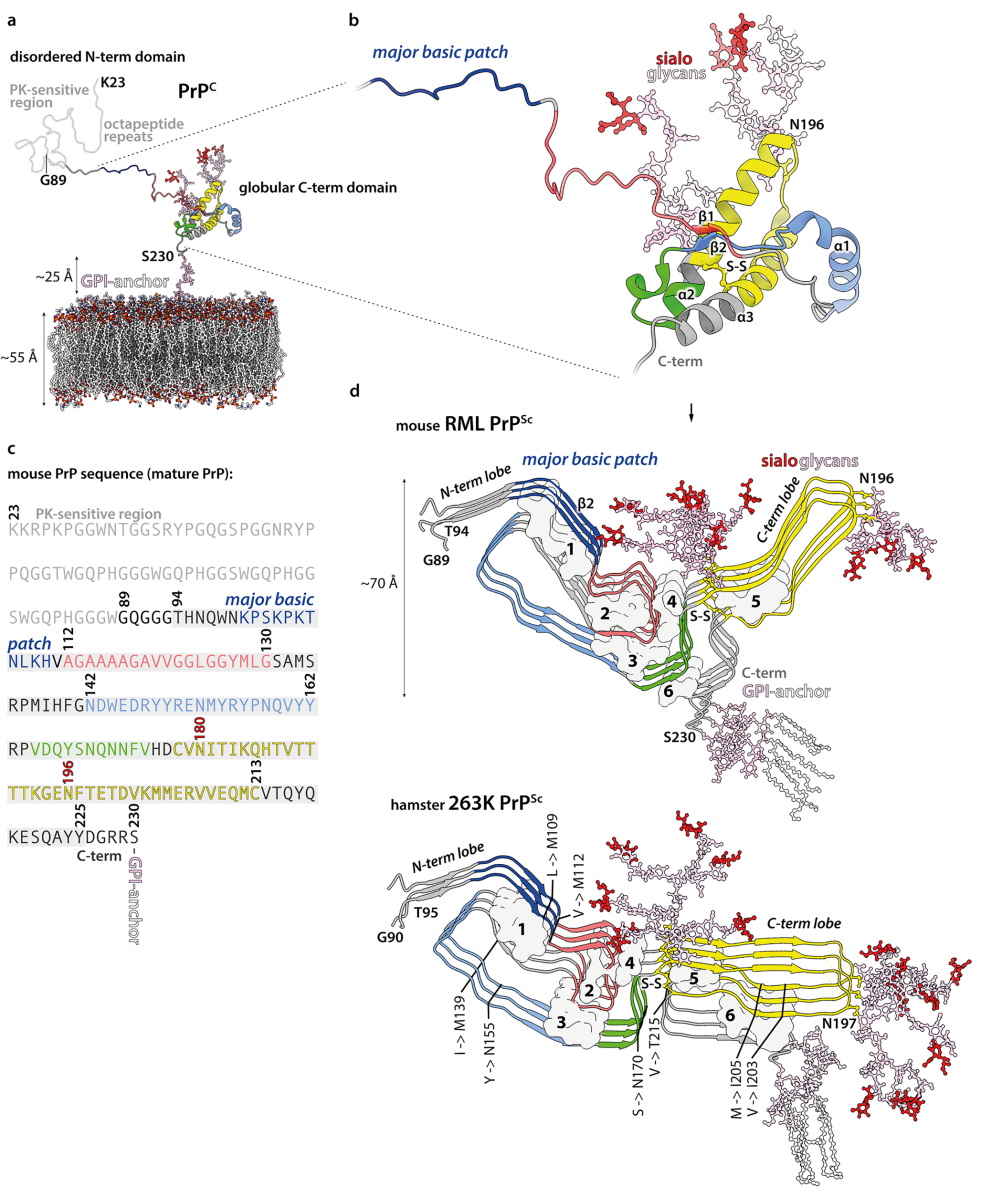
Prion protein conversion into infectious prion fibrils. **a**, **b** Atomistic model of mature mouse PrP^C^ (residues 23–230), including post-translational modifications (carbohydrate groups, pink; sialic acid groups, red), anchored in the phospholipid bilayer (coloured by heteroatom: C, white; P, orange; N, blue; O, red). Proteinase K (PK)-sensitive region (residues 23–89, light grey) refers to PK-sensitivity after conversion to prion fibrils (see panel **d**). The model was built in CHARMM-GUI (https://www.charmm-gui.org/) (Jo et al. 2008) and UCSF Chimera (Pettersen et al. 2004), using a solution NMR structure of the GPI-anchor from human complement regulatory protein CD59 (pdb ID: 1CDR) (Fletcher et al. 1994), an X-ray structure of the mouse PrP (pdb ID: 4H88) (Sonati et al. 2013), and X-ray structures of tri-antennary N-linked sialylated glycans from human prostate specific antigen glycoprotein (pdb ID: 3QUM) (Stura et al. 2011). The close-up view (**b**) shows the portion of the PrP^C^ chain (ribbon representation) that contributes specific sub-domains in PrP^Sc^, as indicated with distinct colours. Selected secondary structures are labelled. **c** Mouse PrP sequence with colour-coded prion fibril PrP^Sc^ sub-domain ranges. PK-resistant RML fibril core (panel **d**, top; residues 89–230) includes amyloid core (residues 94–225; grey highlight). N180 and N196 glycosylation sites are numbered in red. **d** RML (pdb ID: 7QIG) (Manka et al. 2022b) and 263K (pdb ID: 7LNA) (Kraus et al. 2021) PrP^Sc^ fibril structures (3 subunits, ribbon representation) coloured as in (**a**–**b**). N- and C-terminal flexible tails (residues 89/90–93/94 and 226/227–230/231, mouse/hamster numbering) have been added to the models, together with post-translational modifications. The 263K model has additional residues 94–96 modelled at the tips of the C-terminal lobe hairpins, due to their absence in the original structure. Major internal hydrophobic clusters that contribute to fold stability (1–6) are shown with surface representation. Mouse-to-hamster substitutions in PrP sequence are indicated in the 263K structure (hamster numbering). S–S, disulphide bond

PrP monomer incorporation into infectious, protease-resistant, detergent-insoluble fibrillar prion assemblies (classically designated as PrP^Sc^ (Prusiner 1998)) requires gross rearrangement of the protein fold (Fig. 1). While the cellular isoform of PrP (PrP^C^) has globular C-terminal domain containing three α-helices (Wuthrich and Riek 2001; Rodriguez et al. 2017) (Fig. 1a, b), and is soluble in detergents and readily digested by proteases, PrP monomers within detergent-insoluble, infectious PrP^Sc^ fibrils adopt a β-strand-rich configuration (Pan et al. 1993; Prusiner 1998) that confers protease-resistance to the C-terminal two-thirds of the PrP polypeptide (Meyer et al. 1986; Prusiner 1998; Wenborn et al. 2015; Kraus et al. 2021; Manka et al. 2022b; Hoyt et al. 2022) (Fig. 1a and c, d). Proteinase K (PK)-digestion of PrP^Sc^ generates a classical PrP 27–30 banding pattern on SDS-PAGE gels/western blots, comprising C-terminal proteolytic fragments of di-, mono-, and non-glycosylated PrP (Meyer et al. 1986; Prusiner 1998; Wenborn et al. 2015; Kraus et al. 2021; Manka et al. 2022b; Hoyt et al. 2022). The overall similarity of PrP 27–30 banding patterns seen across multiple human and animal prion strains is indicative of a generic fibrillar prion architecture. However, strain-specific signatures of proteolytic fragment sizes and PrP glycoform ratios indicate structural variation in fibrils of different strains (Bessen and Marsh 1994; Telling et al. 1996; Collinge et al. 1996; Parchi et al. 1999; Safar et al. 1998; Hill et al. 2003; Wadsworth et al. 2004; Hill et al. 2006; Wadsworth and Collinge 2011; Wenborn et al. 2015).

**Fig. 2:**
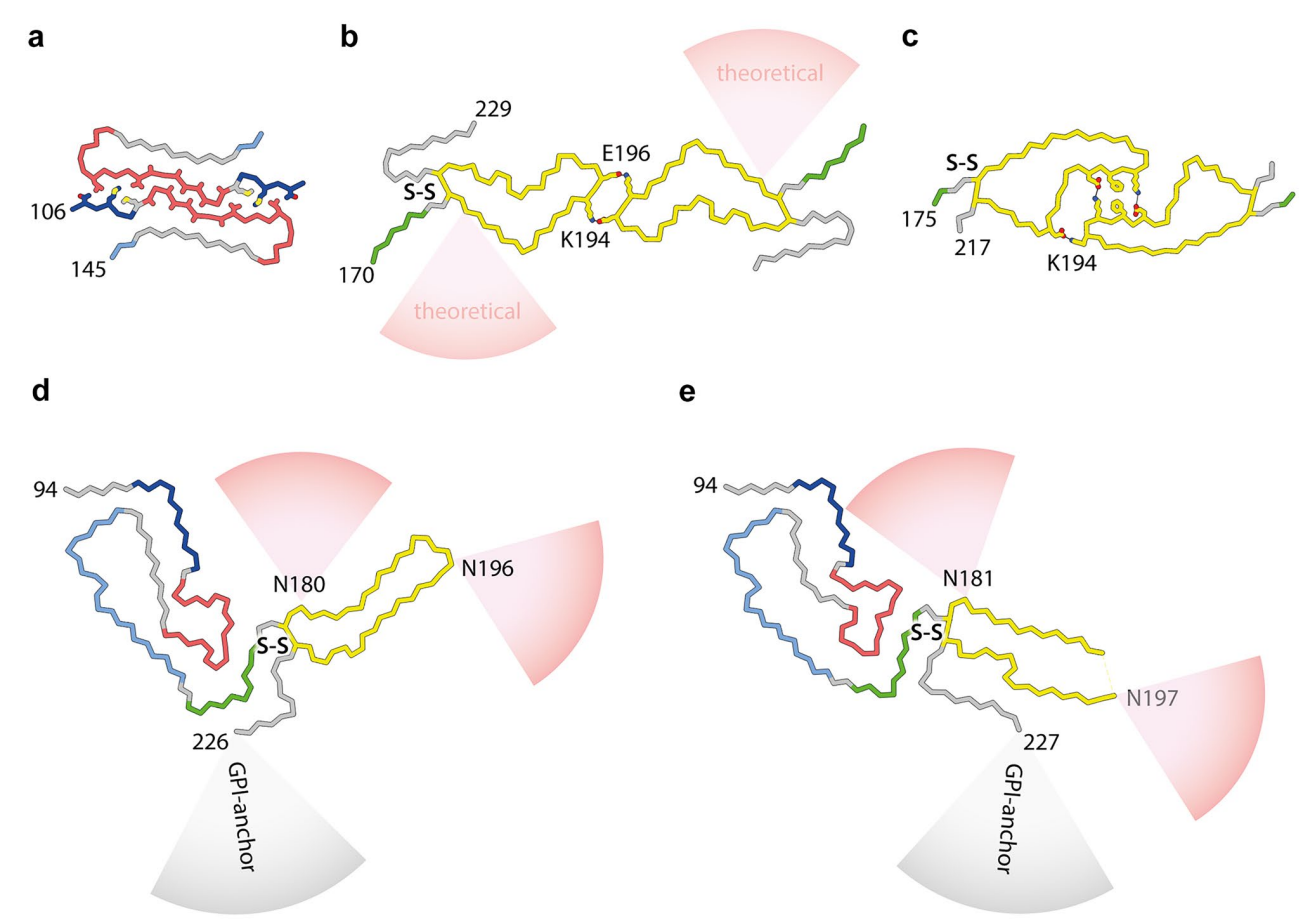
PrP chain conformations in recombinant and ex vivo PrP fibrils **a**–**c** PrP folds and protofilament pairing interfaces in fibrils grown in vitro from recombinantly expressed PrP substrates (human sequence): **a** 106–145 9.7 kDa fragment (M129 variant) (Glynn et al. 2020); **b** 23–231 (full-length) PrP (Wang et al. 2020) with schematically indicated theoretical sialoglycan occupancy (red cones), as modelled by Artikis et al. (2020); **c** full-length PrP with familial prion disease-related mutation E196K (Wang et al. 2021). **d** RML protofibril (Manka et al. 2022b). **e** 263K protofibril (Kraus et al. 2021). Structures in all panels (**a**–**e**) represent fibril cores with species-specific numbering of start and end residues. PrP subunits are shown predominantly as backbones coloured according to ex vivo fibril sub-domain assignment defined in Fig. 1. Residues involved in PrP protofibril pairing (contributing to experimentally confirmed inter-protofilament interfaces) are shown with side chains coloured by heteroatom (N, blue; O, red; S, yellow; C, as backbone) and salt bridges or hydrogen bonds stabilising the two-protofilament architecture are indicated with black lines. Selected interfacing and glycosylated residues are labelled. S–S, disulphide bond

The detailed arrangement of β-strands and overall architecture of infectious ex vivo prion fibrils (also referred to as prion rods (Prusiner et al. 1983; Prusiner 1998; Terry and Wadsworth 2019)) have been intensely debated, reviewed by Rodriguez et al. (2017), Baskakov et al. (2019), and Terry and Wadsworth (2019). Two major structural models have been proposed, the parallel in-register intermolecular β-sheet (PIRIBS) architectures (Groveman et al. 2014; Artikis et al. 2020) and a 4-rung beta solenoid model (Vazquez-Fernandez et al. 2016; Spagnolli et al. 2019). While PrP amyloids formed in vitro from recombinant PrP have PIRIBS architectures (Cobb et al. 2007; Tycko et al. 2010; Groveman et al. 2014; Theint et al. 2017; Wang et al. 2020, 2021; Glynn et al. 2020) (Fig. 2a, c), such assemblies are either devoid of detectable prion infectivity or have such low specific-infectivity that observed structures cannot be reliably correlated with biological activity (Collinge and Clarke 2007; Schmidt et al. 2015; Collinge 2016; Terry and Wadsworth 2019). Efforts to elucidate authentic prion structures have therefore remained reliant upon structural characterisation of ex vivo purified material of high specific infectivity and in a form suitable for structural study.

## Progress with purifying prions

Purification of prions from mammalian brain is highly challenging due to their low abundance in affected tissue and the requirement to maintain and measure biological activity throughout purification. The seminal purification procedures developed by Prusiner and colleagues (Bolton et al. 1982; McKinley et al. 1983; Prusiner et al. 1983, 1987; Bolton and Bendheim 1991) were a remarkable accomplishment and led to the discovery of infectious prion rods (Prusiner et al. 1983). However, the complexity of these procedures and the requirement for large quantities of infected brain has precluded their widespread application and progress with developing alternative purification strategies has been impeded by an historical reliance on rodent bioassay.

The availability of cell-based prion bioassays (Klohn et al. 2003; Mahal et al. 2007) and their automation (Schmidt et al. 2015) has provided the opportunity to explore the development of alternative prion purification strategies. Using the scrapie cell assay (Klohn et al. 2003) to track prion infectivity, we developed novel and simplified procedures for isolating extremely pure, high-titre intact infectious prion rods from small quantities of brain tissue from different hosts (Wenborn et al. 2015). Levels of purity of these samples are comparable with those generated using the best historical protocols, and importantly, as no specialised materials or equipment are required, the method is accessible to most existing prion laboratories. Purified prion rods produced by these methods constitute the authentic infectious prion assembly state as they can be physically correlated with infectivity by bioassay of electron microscopy grids or atomic force microscopy mica supports in cell culture (Terry et al. 2016, 2019) and faithfully transmit prion strain-specific phenotypes when inoculated into mice (Wenborn et al. 2015). These data firmly establish purified prion rods as the target for high-resolution structural investigation of prion strains.

## Progress in cryo-EM and amyloid 3D structure determination

Advances in cryo-EM (Bai et al. 2015; Henderson 2018; Frank 2018; Dubochet 2018; Saibil 2022) and image processing in Relion (He and Scheres 2017; Zivanov et al. 2018; Scheres 2020; Kimanius et al. 2021; Lövestam and Scheres 2022) have enabled direct, high-resolution structural studies of amyloids, fibrillar polymers defined by cross-β structure, in which misfolded protein monomers stack to form a ribbon of intermolecular β-sheets (Eisenberg and Sawaya 2017; Rodriguez et al. 2017; Fitzpatrick and Saibil 2019; Gallardo et al. 2020; Sawaya et al. 2021). In a series of seminal studies using purified fractions from human brains, cryo-EM and image processing in Relion revealed the structures of diverse self-propagating assemblies of tau (Fitzpatrick et al. 2017; Falcon et al. 2018, 2019; Zhang et al. 2020; Shi et al. 2021), amyloid-β (Kollmer et al. 2019; Ghosh et al. 2021; Yang et al. 2022a), and α-synuclein (Schweighauser et al. 2020; Yang et al. 2022b). These remarkable methods and data now lay the foundation for structure-based classification of the commoner neurodegenerative diseases and exploration of the general applicability of prion-like propagation and the strain phenomenon in determining phenotype (Shi et al. 2021; Sawaya et al. 2021; Kovacs et al. 2022).

## Recent cryo-EM structures of ex vivo hamster 263K and mouse RML prion fibrils

High-resolution cryo-EM studies of infectious, brain-derived, prion samples isolated from the hamster 263K prion strain (Kraus et al. 2021) or the mouse RML prion strain (Manka et al. 2022b) discovered single-protofilament, left-handed helical PIRIBS amyloid fibrils that have a remarkably similar overall architecture, despite eight mouse vs. hamster amino acid substitutions (Fig. 1d). In both protofibrils, a single PrP subunit folds to create distinct N- and C-terminal lobes that form each protease-resistant rung of the fibril with protease-resistant cores that correspond to the sequences expected from the strain-specific signature PrP 27–30 truncated PrP^Sc^ banding patterns seen on western blots. GPI-anchorless PrP transgenic mice infected with wild-type RML prions propagate poorly glycosylated prion fibrils (designated aRML) whose fold is very similar to wild-type RML fibrils (Hoyt et al. 2022; Manka et al. 2022b). These findings establish that the wild-type RML fibril fold can be stably propagated utilising mouse PrP lacking post-translational modifications (Hoyt et al. 2022; Manka et al. 2022b).

The N-terminal lobe exhibits more conformational similarity between the 263K and RML strains than the C-terminal lobe that harbours the two N-linked glycans and the GPI anchor (Fig. 1d). Besides the typical longitudinal (inter-chain) cross-β hydrogen bonds in the PIRIBS arrangement, the folds are also stabilised by lateral inter- and intra-chain polar interactions and by six major hydrophobic clusters internal to the fold. These hydrophobic clusters provide both lateral and longitudinal stabilisation to the assembly (Fig. 1d). The overall positions of clusters 1–5 are similar between the two strains; however, cluster 6 is formed by different residues due to divergent C-terminus configuration in each strain (Fig. 1d). This change in C-terminus position likely results from mouse vs. hamster differences in PrP primary structure that map to both (alternative) locations of cluster 6 (Fig. 1d, bottom panel). It also results in the GPI-anchor being much closer to the second (N197) glycosylation site in the 263K fibril, compared to RML fibril (Fig. 1d).

The N- and C-terminal lobes can be further sub-divided into common motifs and folding modules. The first two are the major basic patch (residues 100/101–110/111, mouse/hamster sequence) and the low-complexity Gly/Ala-rich region (residues 112/113–130/131, mouse/hamster sequence), formed from the disordered N-terminal part of PrP^C^ (Fig. 1b and d). The major basic patch (comprising the second β-sheet) shows a relatively conserved topology between the two strains despite two amino acid substitutions, whereas the sequence-wise conserved Gly/Ala-rich region adopts a unique fold in each strain (Fig. 1d).

The major basic patch seems to be firmly stabilised by hydrophobic cluster 1 in both strains (Fig. 1d). Strikingly, the cluster itself is conserved in the two rodent species albeit with three hydrophobic amino acid substitutions (Fig. 1d). In the hamster PrP sequence the L108, V111 and I138 residues (mouse numbering) are replaced with the methionine triad (M109, M112, and M139, hamster numbering) (Kraus et al. 2021; Manka et al. 2022b). The relatively long, inwardly pointed Met side chains (Kraus et al. 2021) considerably widen the respective β-hairpin structure (Fig. 1d). Notably, a single M for I substitution at the position 138 (mouse numbering) was found to be inhibitory for scrapie prion propagation in mouse neuroblastoma cells in 1995 (Priola and Chesebro 1995). Now, after 27 years, we can appreciate how this single amino acid mismatch may cause a prion transmission barrier, at least in this particular cell-based system.

Hydrophobic clusters 2 and 3 stabilise variable conformations of the low-complexity Gly/Ala-rich region within the N-terminal lobe, whereas hydrophobic cluster 4 provides this region with additional hydrophobic anchorage at the inter-lobe interface in both protofibrils (Fig. 1d). On the other side of the N-terminal lobe, the sequence 142/143–162/163 (mouse/hamster numbering) (which in PrP^C^ forms the first α-helix (α1), the second (short) β-strand (β2) and the intervening α1-β2 loop (Fig. 1b)) shows a similarly folded region in both fibril structures (Fig. 1b and d). It is almost immediately followed by the sequence 165/166–175/176 (mouse/hamster numbering), which in PrP^C^ comprises the β2-α2 loop (Fig. 1b), that folds to create the C-terminal side of the inter-lobe interface and interacts with the Gly/Ala-rich region in both fibrils (Fig. 1b and d).

The disulphide-stapled hairpin is a major part of the C-terminal lobe in both strains and shows a distinct conformation in each strain, which requires unravelling α-helices 2 and 3 of PrP^C^. Unlike the RML disulphide-stapled hairpin, the 263K hairpin widens towards its tip, presumably due to the unique, hydrophobic cluster 6-mediated interaction with the C-terminus, as described above (Fig. 1d). This 263K-specific interaction may also be responsible for widening of the cleft between the N- and C-terminal lobes in the 263K fibril compared to the RML fibril, which potentially reduces spatial constraints for the N180-glycan in the former compared to the latter (Figs. 1d and 2d, e).

Overall, the PIRIBS structure of the three ex vivo prion fibrils characterised to date (Kraus et al. 2021; Manka et al. 2022b; Hoyt et al. 2022) is compatible with earlier studies that examined ex vivo material using a variety of other techniques (reviewed in Kraus et al. (2021)) and with the defining physicochemical properties of prions (Kraus et al. 2021; Manka et al. 2022b; Hoyt et al. 2022). These authentic infectious prion fibrils appear to incorporate particularly long polypeptide chains into their amyloid cores compared to the other disease-related amyloids that have been characterised so far (Sawaya et al. 2021; and see The Amyloid Atlas https://srv.mbi.ucla.edu/AmyloidAtlas/), and such unique fibril structures, stabilised by cohorts of internal hydrophobic clusters spanning the entire length and width of the fibrils, may explain the exceptional stability of prions and their resistance to clearance by the host.

## Cryo-EM structures of recombinant PrP fibrils

To date, no in vitro-generated fibrils polymerised from recombinant (anchorless and strictly non-glycosylated) PrP monomers have replicated the structures of ex vivo prion fibrils. All recombinant PrP fibrils so far characterised by cryo-EM consist of two symmetrical protofilaments with smaller amyloid cores than those of ex vivo infectious prion fibrils (Wang et al. 2020, 2021; Glynn et al. 2020) (Fig. 2). None of these structures have been reported to be infectious (Wang et al. 2020, 2021; Glynn et al. 2020).

Notably, the recombinant PrP fibrils show various geometries and pairing interfaces (Wang et al. 2020, 2021; Glynn et al. 2020) (Fig. 2a–c). Such pairing promiscuity appears to be only possible for the relatively small, non-glycosylated amyloid cores of recombinant PrP fibrils, as the observed pairing modes are largely incompatible with the ex vivo fibril folds or the locations of N-linked glycans (Fig. 2d, e).

## Paired prion fibrils

Consistent with previous studies (Terry et al. 2016, 2019), our recent high-resolution cryo-EM studies showed that RML protofibrils can associate laterally, forming two-protofilament fibrils (Manka et al. 2022b) (Fig. 2d). However, analogous paired assemblies of 263K protofibrils were not reported (Kraus et al. 2021). The reason for this difference is currently unclear, and in this regard, we do not know how different prion extraction and purification protocols used in our work and that of Caughey and colleagues might influence protofilament pairing. Kraus et al. used high salt concentrations (1.7 M NaCl) (Kraus et al. 2021), and we used phosphotungstate (PTA) polyanions that clearly decorate RML fibrils along the major basic patch and in the vicinity of the inter-protofilament interface of the predominant paired fibril structure identified in our cryo-EM dataset (Manka et al. 2022b) (EMPIAR-10992). In the absence of a high-resolution 3D reconstruction, it cannot be excluded that PTA clusters are capable of bridging two RML protofibrils together or modulating existing paired fibril geometries. Indeed, more than one mode of RML protofibril pairing was detected in our cryo-EM dataset (Manka et al. 2022b) (EMPIAR-10992). However, crucially, we confirmed that pairing per se is independent of PTA, as paired protofilaments are also present in ex vivo RML prion preparations that excluded PTA (Manka et al. 2022b).

## Potential mechanisms underlying prion-strain specific PrP glycoform ratios

Not all prion strains are considered to be PrP sialoglycoform-selective. The hamster 263K strain is an example of such non-selective strain, whose PrP sialoglycoform composition appears to reflect the natural (physiological) abundance of PrP^C^ sialoglycoforms, in which di-glycosylated PrP is dominant (Katorcha and Baskakov 2017). In contrast, the RML strain has diminished di- and enriched mono- and non-glycosylated PrP composition (Wenborn et al. 2015; Katorcha and Baskakov 2017; Kang et al. 2020; Manka et al. 2022b). Thus, the architecture of the RML prion is expected to sterically restrict incorporation of di-glycosylated PrP chains and favour incorporation of mono- and non-glycosylated PrP. On the basis of the existing data, it is not readily apparent how this could be achieved based solely on the single protofilament architecture. Indeed, in silico modelling suggests no obvious steric hindrance or energetic penalty for accommodating even large tri-antennary sialoglycans at every rung of the PIRIBS structure of a recombinant PrP fibril (Artikis et al. 2020) (shown in Fig. 2b). Paired assemblies of RML protofilaments, however, could confine the space available for N-glycan incorporation (Manka et al. 2022b). Clearly further work on the paired assemblies is required and such future work may establish whether protofilament pairing is a specific property of glycoform selective strains or a universal phenomenon across prion strains.

## Prions versus transmissible PrP amyloid

Human prion diseases are associated with a range of clinical presentations, and they are classified by both aetiology and clinicopathological syndrome, with subclassification according to molecular criteria (Collinge et al. 1996; Collinge 2001; Parchi et al. 1999; Hill et al. 2003; Wadsworth and Collinge 2011; Baiardi et al. 2018; Mead et al. 2019). Approximately 15% of cases are associated with autosomal dominant pathogenic mutations in the human prion protein gene (*PRNP*), and to date, more than sixty mutations have been described (Mead et al. 2019). These include insertions of between four and twelve extra repeats within the octapeptide repeat region (Fig. 1a) between codons 51 and 91, a two-octapeptide repeat deletion and various other mutations causing missense or stop substitutions or other insertions with and without a frameshift (Mead et al. 2019).

Pathogenic *PRNP* mutations appear to have diverse and direct effects on dictating the preferred structure or assembly state of mutant PrP prion assemblies and cases of inherited prion disease caused by point mutations have PrP 27–30 glycoform ratios distinct from those seen in both sporadic and acquired CJD human prion diseases (Piccardo et al. 1998; Parchi et al. 1998; Furukawa et al. 1998; Cardone et al. 1999; Hill et al. 2006). Moreover, in distinction to sporadic or acquired CJD, a common feature in patients with diverse *PRNP* point mutations is that the expressed full-length mutant PrP generates two distinct types of disease-related PrP assembly. One corresponds to authentic prions that generates classical PrP 27–30 patterns of N-terminally truncated PrP^Sc^ and is found in brain areas showing synaptic PrP deposition, spongiform vacuolation, and neurodegeneration. The other type of assembly forms smaller N- and C-terminally truncated protease-resistant fragments (typically 7–15 kDa, derived from the central region of PrP) and is associated with prominent PrP amyloid plaques that are commonly seen in some inherited prion diseases (Giaccone et al. 1992; Piccardo et al. 1996, 1998, 2001; Parchi et al. 1998; Salmona et al. 2003; Hill et al. 2006; Wadsworth et al. 2006; Monaco et al. 2012; Asante et al. 2013, 2020; Ghetti et al. 2018; Cracco et al. 2019). Variation in the PrP conformation of authentic prion fibrils and distinct PrP amyloids (governed by the specific PrP missense mutation) would be expected to dictate highly specific strain transmission properties via conformational selection (Collinge 1999, 2016; Collinge and Clarke 2007; Wadsworth et al. 2010) as has recently been demonstrated for *PRNP* P102L and A117V mutations (Asante et al. 2009, 2013, 2015, 2020).

Importantly, temporal and spatial differences in the propagation of authentic prions or alternative PrP amyloids within the brain may readily account for the wide diversity of clinicopathological phenotypes seen in family members with the same *PRNP* mutation (Parchi et al. 1998; Kovacs et al. 2002; Piccardo et al. 2007; Webb et al. 2008; Mead and Reilly 2015; Barron et al. 2016; Choi et al. 2016; Barron 2017; Kim et al. 2017; Ghetti et al. 2018; Mead et al. 2019; Asante et al. 2020) with further complexity contributed by variable involvement of pathological conformers of wild type PrP (Gabizon et al. 1996; Silvestrini et al. 1997; Chen et al. 1997; Wadsworth et al. 2006; Monaco et al. 2012). Notably, while patients with different *PRNP* missense mutations that produce full length mutant PrP can co-propagate authentic prions and alternative PrP amyloids, patients with *PRNP* stop mutations which produce C-terminally truncated PrP devoid of N-linked glycans and GPI-anchor (for example, Y145X (Ghetti et al. 1996) or Y163X (Mead et al. 2013)) may only propagate PrP amyloids giving rise to distinct disease phenotypes (Mead and Reilly 2015; Ghetti et al. 2018). To date, high-resolution structural studies of ex vivo prions and alternative PrP amyloids from human brain have been extremely problematic due to biosafety obstacles; however, very recently, cryo-EM structures of prion protein filaments from Gerstmann-Sträussler-Scheinker disease (F198S) have been reported (Hallinan et al. 2022).

## Progress in defining the neurotoxic PrP species

Prion pathogenesis involves progressive loss of neuronal cells, and it has been long assumed that prions are directly neurotoxic. However, using a new multi-parametric assay of prion neurotoxicity in primary neurons, we recently examined exceptionally pure preparations of highly infectious, ex vivo RML prion fibrils devoid of contaminating proteins or small oligomeric PrP assemblies in cell culture alongside RML prion-infected mouse brain homogenates. Prion-infected brain homogenates were toxic as measured by neurite retraction and fragmentation and reduction in dendritic spine density (Benilova et al. 2020). In sharp contrast, highly purified infectious prion fibrils (100–200 nm in length) were not toxic and did not induce neurite retraction even when at prion titres much higher than that present in the infected brain homogenate (Benilova et al. 2020). Significantly, treatment of brain homogenate with the detergent sarkosyl abolished neurotoxicity, while infectivity was unaffected. These data clearly suggest that neurotoxicity is not attributable to infectious prion assemblies regardless of their aggregate size or relative protease sensitivity (Benilova et al. 2020). The lack of detectable direct toxicity of highly purified prion preparations or sarkosyl-treated infected brain homogenate is consistent with models of prion neurotoxicity being mediated by a distinct oligomeric or monomeric PrP isoform designated PrP^L^ (for lethal) that may be generated by a distinct mechanistic process (Collinge and Clarke 2007; Sandberg et al. 2011, 2014; Collinge 2016; Benilova et al. 2020).

## Concluding remarks

Undoubtedly, we have just entered a very exciting era for prion research, where methods for prion fibril purification and near-atomic structure determination by single particle cryo-EM are established (Telling 2022). A flurry of ex vivo prion fibril structures can now be expected (see for example Manka et al. (2022a)). Defining prion replication mechanisms responsible for maintaining the signature glycoform ratios of diverse prion strains remains a considerable research challenge, and it now seems clear that determining the relevance of paired fibril assemblies may be critical to achieving this. Understanding mechanisms of neurotoxicity in prion disease pathogenesis remains highly challenging; however, new neurotoxicity assays are now enabling systematic isolation of the neurotoxic PrP species that will facilitate detailed structural characterisation (Benilova et al. 2020). Future microscopy-based research is now also likely to move toward high-resolution studies of prion interactions with their cellular or extracellular binding partners and in this regard several extracellular matrix proteins or cell surface receptors have been implicated in prion pathogenesis (Moretto et al. 2022). Correlative light and electron cryo-tomography involving fluorescent labelling of PrP in a way that does not interfere with prion propagation will be necessary for facilitating this future research. This would then permit identification of relevant prion structures within the complex environments of cell cultures or tissue sections.
